# Economic development and emotional well-being: longitudinal evidence from 33 European countries

**DOI:** 10.1038/s41598-026-59695-1

**Published:** 2026-07-05

**Authors:** Filip Fors Connolly, Mikael Hjerm, Tommy Gärling

**Affiliations:** 1 Department of Sociology, Umeå University, Umeå, Sweden; 2 Department of Psychology, Stockholm University, Stockholm, Sweden; 3 Department of Psychology, University of Gothenburg, Göteborg, Sweden

**Keywords:** Economic development, Emotional well-being, Life satisfaction, Cross-national comparison, European Social Survey, Multilevel modeling, Health care, Psychology, Psychology

## Abstract

The relationship between economic development and emotional well-being is less well understood than the corresponding relationship with life satisfaction, partly because prior cross-national studies have often relied on brief dichotomous affect measures. Using four waves of the European Social Survey (2006, 2012, 2014, and 2023), we aggregated responses from 177,948 respondents in 33 countries to 97 country-round observations and linked these observations to real GDP per capita. We found strong positive associations between GDP and both emotional well-being and life satisfaction across countries. Longitudinal mixed models showed positive associations between GDP and both well-being components over time. Supplementary checks using separate affect components, quadratic GDP specifications, demographic-composition controls, contextual adjustment for social trust and income inequality, attenuation models with perceived income adequacy and unemployment, and growth-versus-decline tests broadly supported this pattern. Overall, the findings challenge previous conclusions that economic development is only weakly related to affect. When emotional well-being is measured with graded content-valid affect items, it shows a clear positive relationship with both GDP levels and changes in GDP.

**Supplementary Information:**

The online version contains supplementary material available at 10.1038/s41598-026-59695-1.

## Introduction

 The relationship between economic development and subjective well-being has been debated for five decades^1,2^. Although extensive research has examined life satisfaction and generally finds a positive association between economic development and life satisfaction^3,4^, the relationship between economic development and emotional well-being remains less well understood. This gap in knowledge is particularly significant given evidence that emotional well-being may be more important than life satisfaction for tracking people’s quality of life and welfare^5^ and that different components of well-being may be influenced differently by economic resources^6^. The aim of this study is to investigate how national income levels are longitudinally associated with emotional well-being.

Life satisfaction refers to a global evaluative judgment in which respondents integrate information about their circumstances, aspirations, standards, and perceived life trajectory. Emotional well-being, by contrast, refers to the affective quality of experience, commonly operationalized as the frequency or duration of positive and negative affect over a specified period^7^. Thus, although life satisfaction and emotional well-being are often assessed using different temporal frames, the distinction between them is not only temporal; life satisfaction captures evaluative judgments about life as a whole, whereas emotional well-being captures the affective balance of everyday experience. While these two components of subjective well-being are positively correlated, they also exhibit unique variances, suggesting that they are not interchangeable^6,8^. In fact, many scholars argue that experiences of enjoyment and suffering carry greater moral and political weight than abstract evaluations^5,9^. This distinction is vital for policy discussions. If higher GDP per capita is associated with emotional well-being alongside life satisfaction, it makes a stronger case for the relevance of economic development than if GDP merely predicts more favorable abstract life evaluations without corresponding differences in actual daily experiences.

Different predictions can be made of how economic development is related to emotional well-being versus life satisfaction. Although a strong positive cross-sectional correlation between life satisfaction and emotional well-being might suggest similar determinants^10^, theoretical arguments offer competing predictions. According to one line of thinking, economic development mainly raises life satisfaction, with limited association with emotional well-being, because cognitive judgments incorporate long-term opportunities and material resources^6^ whereas affect adapts quickly to improved conditions^11^ or is anchored in stable personality traits^12^. However, higher national income could also be associated with emotional well-being by reducing daily stressors, widening access to enjoyable activities, and reducing financial insecurity^13^. Our analysis investigates how GDP per capita levels and within-country deviations in those levels are associated with emotional well-being, systematically comparing these associations with the well-documented relationship between economic development and life satisfaction.

This study analyzes high-quality cross-country panel data from the European Social Survey (ESS). The European context provides a valuable setting for our analysis, as European countries constitute a relatively homogeneous set of developed economies with comparatively similar institutional and cultural frameworks, reducing confounding factors often present in global analyses. By employing a multi-item emotional well-being scale with cross-national validity, we enhance the overall measurement quality compared to previous comparative studies, providing more robust and accurate assessments of emotional well-being.

We examine whether economic development is positively associated with emotional well-being both cross-sectionally and longitudinally, and compare the size of this association with the relationship between economic development and life satisfaction. Previous studies have often relied on dichotomous affect measures with questionable content-validity and have reported negligible or non-significant GDP associations for emotional well-being. Our analyses use multi-item affect measures with graded response options and prior evidence of cross-national validity. This design tests a clear measurement-based proposition: GDP-EWB associations may be underestimated when emotional well-being is measured with brief dichotomous yesterday-affect items rather than with more graded, content-valid affect measures.

## Previous research

Cross-national research on subjective well-being has predominantly utilized single-item measures of life satisfaction or global happiness (e.g., “How happy are you?”). These are treated as evaluative indicators because they prompt respondents to make a global assessment of their life as a whole. In contrast, emotional well-being encompasses affective experiences over a specific time frame^14^. This component is commonly assessed by asking respondents about the frequency of positive and negative emotions experienced during a recent period, such as yesterday or the past week.

The relationship between economic growth and subjective well-being gained prominence with Easterlin’s^1^ seminal observation that despite substantial economic growth in the United States, average levels of life satisfaction showed no clear upward trend. This finding, later termed the Easterlin Paradox, suggested that beyond meeting basic needs, increases in national income do not translate into improvements in subjective well-being. The Easterlin Paradox has sparked considerable debate and empirical investigation. Several mechanisms have been proposed to explain why economic growth seems to fail to improve well-being. Social comparison theory suggests that well-being depends not on absolute income but on relative position within society^15^. If economic growth preserves or even increases inequality, the well-being gains from higher absolute income may be offset by unchanged or worsened relative position. Adaptation theory proposes that individuals adjust to improved circumstances, returning to baseline well-being levels despite permanently higher incomes^16^.

Numerous studies challenge the Easterlin Paradox using global measures of life satisfaction and happiness. Deaton^17^, using Gallup World Poll (GWP) data, found a strong positive cross-country link between logged GDP per capita and life satisfaction. This effect held across all income levels, with no diminishing returns and possibly stronger effects in wealthy nations, suggesting a robust association with subjective well-being even at high income. Further evidence from Stevenson and Wolfers^18,19^ and Sacks et al.^2^, analyzing multiple large-scale, long-term international datasets, established consistent positive associations between subjective well-being and GDP. Their work showed a consistent log income gradient across individuals, countries, and over time, highlighting the importance of increases in absolute income with no saturation point. Inglehart et al.^20^, using World Values Survey data (1981–2007), also found positive relationships between life satisfaction and national income, with upward happiness trends alongside development in most countries. Analyses with a particular focus on extensive time-series data offer more direct rebuttals of Easterlin’s assertion of no long-term growth-happiness correlation. For example, Veenhoven and Vergunst^21^ used extensive data (1531 data points, 67 nations, 10–40 years) and found a positive correlation between GDP growth and rising subjective well-being (life satisfaction and happiness). Cai et al.^22^ showed a similar upward happiness trend with China’s rapid economic growth. Recent European studies find the same pattern. Frech et al.^23^, analyzing 33 European countries (2002–2018), found that GDP per capita is associated with life satisfaction both between countries and within countries, particularly for the 10% worst-off, affirming a robust growth link with life satisfaction even in wealthy European nations.

While the evidence consistently supports a positive overall relationship between economic growth and life satisfaction, several studies highlight potential nuances, methodological issues, and moderating factors. For example, in a time-series study of ten European nations from the early 1980s to 2018, Easterlin and O’Connor^24^ found the relationship between long-run changes in happiness and economic growth to be statistically insignificant. Their analysis concluded that these happiness trends were instead principally determined by the generosity of welfare state programs. Mikucka et al.^3^ emphasized that the relationship between economic growth and life satisfaction varies considerably depending on institutional and social contexts. Their analysis revealed that economic growth unconditionally improved life satisfaction in transition economies but conditionally, dependent on increased social trust and reduced income inequality, in non-transition countries. Further, Bartolini and Sarracino^25^ demonstrated variability in the correlation between GDP and life satisfaction depending on the time horizon; stronger correlations appeared in the short term but diminished over medium to long-term periods. Despite these nuances, the overarching conclusion from robust cross-national and longitudinal evidence is that higher GDP per capita is reliably associated with increased life satisfaction and happiness, although debates about the precise long-term effects continue due to the limited availability of very long time-series data for a large set of countries.

A few studies have examined economic development and emotional well-being using explicit measures of affect in the Gallup World Poll (GWP). For example, Diener et al.^26^ found substantially stronger cross-sectional correlations between GDP per capita and life satisfaction (*r* = .78) than positive affect (*r* = .29) and negative affect (*r* = − .04) between 2005 and 2011. In addition, the longitudinal analysis showed that while GDP per capita predicted changes in life satisfaction over time, it did not predict changes in positive or negative affect. Further assessing longitudinal associations in GWP data, Diener and Tay^27^ reported a significant, albeit modest, positive correlation between changes in GDP and changes in life satisfaction between 2006 and 2013 (*r* = .08). For specific emotions, their study indicated that changes in GDP had non-significant correlations with changes in enjoyment (*r* = − .02) and anger (*r* = − .06) but were significantly negatively correlated with changes in sadness (*r* = − .10) and stress (*r* = − .11). Consistent with the general pattern of a stronger association between economic development and life evaluations than with emotional well-being, large-scale panel data analyses by Helliwell et al.^28^, employing pooled OLS regressions on GWP data (2005–2018), found that log GDP per capita was a strong and positive predictor of life evaluations, while it was not significantly associated with positive affect and negative affect. These findings suggest a fundamental difference in how economic development relates to life satisfaction versus emotional well-being. However, the reliability of previous studies is reduced by several methodological concerns that, so far, have received limited attention.

First, emotional well-being in the GWP is assessed using composite measures constructed from dichotomous survey items (“yes/no” questions about emotional experiences yesterday), whereas life satisfaction is measured with an 11-point numeric scale. This difference in measurement scales could reduce the observed relationship between GDP and emotional well-being due to the lower variance and sensitivity of dichotomous measures compared to numeric scales. Evidence supporting this concern comes from individual-level research. Killingsworth^29^ found that when emotional well-being was assessed by means of experience sampling using a continuous sliding scale, no saturation point in the relationship with income was observed. In contrast, a saturation point emerged when emotional well-being was measured using dichotomous items identical to those in the GWP, suggesting that the response scale significantly impacted the observed associations.

Second, some emotional well-being items in the GWP have questionable content validity for capturing the valence of affective experiences. For example, items like “Did you laugh or smile a lot yesterday?” may not adequately represent the valence of emotional states, as individuals can experience positive emotions without necessarily expressing them through overt behaviors like smiling or laughing. This concern is particularly critical across different cultural contexts, where emotional expression norms vary significantly^30,31^. Similarly, the item “Did you experience anger a lot yesterday?” is problematic for measuring negative affect, as anger can serve adaptive functions and may not always be experienced as purely negative, particularly when it motivates corrective action^32^.

Third, previous studies have typically analyzed positive and negative affect separately rather than estimating an overall affect balance measure. While separate analyses are informative for identifying if economic development is more strongly related to pleasant or unpleasant emotional experiences, they do not directly assess whether economic development is associated with the overall affective quality of daily life. This limits comparability with life satisfaction, which is analyzed as a broad global indicator. This approach may also obscure associations that operate in opposite directions across affect components.

Fourth, the GWP assesses emotional experiences with reference to the previous day, which makes the measures particularly sensitive to short-term fluctuations and situational factors. While country aggregation mitigates idiosyncratic individual variation, it cannot eliminate systematic distortions from shared experiences, such as major news events, holidays, unusual weather, or other fieldwork-period effects that may affect entire populations without reflecting more stable well-being conditions. The volatility of a one-day snapshot is especially consequential in longitudinal analyses, where small to moderate within-country changes over time are central to the research question.

Fifth, these methodological limitations become particularly critical when comparing countries with vastly different cultural norms and developmental stages, especially if measurement equivalence has not been established. The global scope of the GWP encompasses nations ranging from subsistence economies to advanced post-industrial societies. Across these diverse contexts, norms surrounding emotional expression and interpretations of survey questions may differ substantially. Without evidence of measurement invariance, observed cross-national variations in the relationship between GDP per capita and emotional well-being risk reflecting measurement artifacts rather than genuine cultural or developmental differences.

Given these methodological concerns in previous studies, the aim of this study is to analyze the relationship between economic development and emotional well-being, both cross-sectionally and over time, using psychometrically adequate measures. We use multi-item scales measuring emotional well-being with graded response options that are high in content-validity and include items that have been cross-country validated in prior research^33,34^. To enable direct comparison with previous research on life satisfaction, we analyze a single-item measure of life satisfaction. This comparative approach allows us to assess the extent to which these different indicators of subjective well-being converge or diverge in their relationships with economic development, providing more reliable evidence on whether higher national income is associated with daily emotional experiences alongside cognitive life evaluations. The period covered (2006–2023) encompasses significant economic upheaval, including the 2008 financial crisis, the COVID-19 crisis, and recent inflationary pressures, providing variation in economic conditions that can illuminate the relationship between economic development and subjective well-being over time.

## Methods

### Data

We used four waves of interview data from the ESS, a biennial cross-national survey of social attitudes, beliefs, and behavior patterns across Europe. We analyze waves 3 (2006), 6 (2012), 7 (2014), and 11 (fielded in 2023/24 and coded as 2023 in the analytic panel). The selected waves include the emotional well-being items required for our analysis. ESS samples are drawn with probability-based methods intended to represent the resident population aged 15 and over in each participating country. Individual responses were aggregated to 97 country-round observations from 33 countries, using the ESS post-stratification weight (pspwght) for all ESS-based country-round means. The analytic respondent sample comprised 177,948 participants with valid life satisfaction or at least one valid emotional well-being item.

### Measures

All four waves of ESS included the CES-D 8 scale for measuring depressive symptoms^35^. Participants indicated how often they experienced different emotional states during the past week using four response options: none or almost none of the time (1), some of the time (2), most of the time (3), all or almost all the time (4). Recent psychometric work using the Swedish ESS CRONOS-2 Panel reveals that the CES-D 8 factor correlates − 0.75 with an independent emotional well-being factor measured on a separate occasion and fails the Fornell-Larcker discriminant-validity test against the same factor^36^. These findings support using selected CES-D affect items as indicators of general emotional valence in this context, while the scale’s origin as a depressive-symptom battery should be kept in mind.

**Fig. 1:**
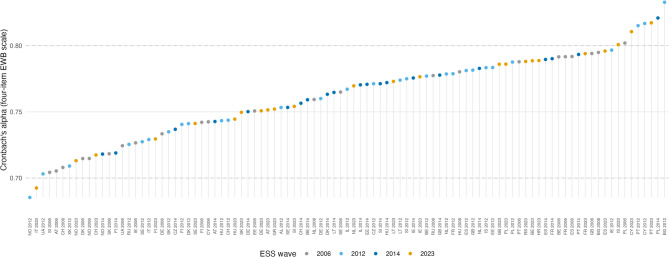
Cronbach’s alpha for the four-item emotional well-being scale by country-round. Each point is the country-round Cronbach’s alpha for the four-item EWB scale (happy, enjoyed life, felt sad, felt depressed; negative items reverse-scored), computed at the individual level on the relevant ESS wave. Country-rounds are ranked from lowest to highest alpha and colored by wave; dashed reference lines mark the conventional thresholds of 0.70 and 0.80.

To measure emotional well-being, we selected four affect items: happy, enjoyed life, sad, and depressed. These items are included in the approximate invariant (or partially invariant) solutions of two recent studies assessing cross-country measurement invariance of affect items in the ESS^33,34^. While those authors retained some additional CES-D 8 items for negative affect, our choice represents a balanced, four-item core designed to maximize cross-national comparability. This core covers both emotional valence poles, possesses high content-validity, and maintains symmetric positive/negative wording. Reliability of the emotional well-being scale was acceptable across country-rounds. Cronbach’s alpha for the four-item EWB scale ranged from 0.685 to 0.833 across the 97 country-rounds (Fig. 1), values that indicate acceptable to good internal consistency for a short cross-national affect scale.

Life satisfaction was assessed using the question: “All things considered, how satisfied are you with your life as a whole nowadays?” Participants responded on a scale ranging from 0 to 10 with endpoints labeled “Extremely dissatisfied” and “Extremely satisfied”. Although this is a single-item measure, it has demonstrated satisfactory convergent validity with multi-item scales and is widely used in cross-national research^37^.

GDP per capita was measured using the World Bank World Development Indicators series for real GDP per capita, PPP, in constant 2021 international dollars (NY.GDP.PCAP.PP.KD). Values were matched to the calendar year preceding each ESS wave (2005, 2011, 2013, and 2022, respectively). Round 11 fieldwork took place in 2023/24, but 2022 is used as a common prior-year exposure for the Round 11 wave to avoid assigning different macro years within the same ESS round. We logarithmically transformed GDP per capita to reflect diminishing marginal utility of income and to reduce positive skewness.

Additional country-round covariates were used in robustness and exploratory attenuation analyses. Demographic composition was measured from the ESS as the post-stratification-weighted percentage of female respondents, mean age, and mean years of education in each country-round. Generalized social trust was measured using the ESS 0–10 trust item and aggregated to the country-round level. Income inequality and unemployment were taken from the World Bank World Development Indicators as the Gini coefficient and unemployment rate. Perceived household income adequacy was measured with the ESS household income item (hincfel), which asks respondents how they feel about their household’s income nowadays. The item was reverse-coded and aggregated so that higher values indicate greater perceived income adequacy. Table A1 reports descriptive statistics for the analytic panel.

### Analysis plan

For clarity and ease of interpretation, we employed 0–10 scales for both life satisfaction and emotional well-being as our primary outcome measures. Life satisfaction was directly measured on this scale. Emotional well-being was constructed from the four selected affect items after reverse-scoring sad and depressed; for respondents with at least one valid selected affect item, the individual score was computed as the mean of available items and then linearly transformed onto a 0–10 scale. Among respondents contributing to the emotional well-being measure, 97.8% had valid responses on all four affect items, so scoring based on the mean of available items affected only a small minority of cases. Country-round emotional well-being means were then computed using the ESS post-stratification weight.

Cross-sectional analyses explored relationships between log real GDP per capita and both dimensions of subjective well-being using scatterplots and correlation analyses. We also examined the direct country-round association between life satisfaction and emotional well-being to assess their overlap.

Longitudinal analyses focused on country-round observations nested within countries. We examined within-country deviations from each country’s mean GDP and well-being, and then estimated mixed models to test whether GDP differences between countries and within-country GDP deviations over the observed waves were associated with emotional well-being and life satisfaction. The analytic panel comprised 97 country-round observations from 33 ESS countries across four ESS waves. Because the waves are unevenly spaced, the time indicators are treated as discrete survey-wave contrasts with 2006 as the reference category.

To differentiate within-country deviations over the observed waves from between-country differences, we decomposed log GDP per capita into two Mundlak components. The between-country component captures each country’s average log GDP over observed waves. The within-country component captures deviations from that country mean. Both the emotional well-being and life-satisfaction models used a random country intercept and an AR(1)-type residual structure over survey-wave order. This residual correlation is indexed by observed wave order rather than calendar-year spacing, treating the four survey waves as equal lags. We used the same residual specification for both outcomes so the GDP coefficients are compared under a common covariance specification. With only four unevenly spaced waves, the AR(1)-type structure should be interpreted as an approximate adjustment for serial dependence rather than a precise continuous-time process; alternative calendar-year residual structures gave near-zero residual autocorrelation and lower fit, so the wave-index version was retained as a pragmatic covariance adjustment.

In response to peer review, we conducted supplementary analyses. These included separate positive- and negative-affect models; tests for asymmetric associations during GDP rises versus declines; quadratic GDP specifications; adjustment for aggregate demographic composition; contextual adjustment for social trust and Gini; and exploratory attenuation models adding perceived income adequacy and unemployment.

During the preparation and revision of this manuscript, the authors used large language model-based AI tools (ChatGPT, OpenAI; Claude, Anthropic) to assist with language editing, proofreading, manuscript consistency checks, and R code writing, checking, and debugging. These tools were not used to generate data or make autonomous analytical decisions. All AI-assisted text, code, analyses, and outputs were reviewed, verified, and approved by the authors, who take full responsibility for the content of the manuscript.

## Results

### Cross-sectional relationships between economic development and well-being

**Fig. 2:**
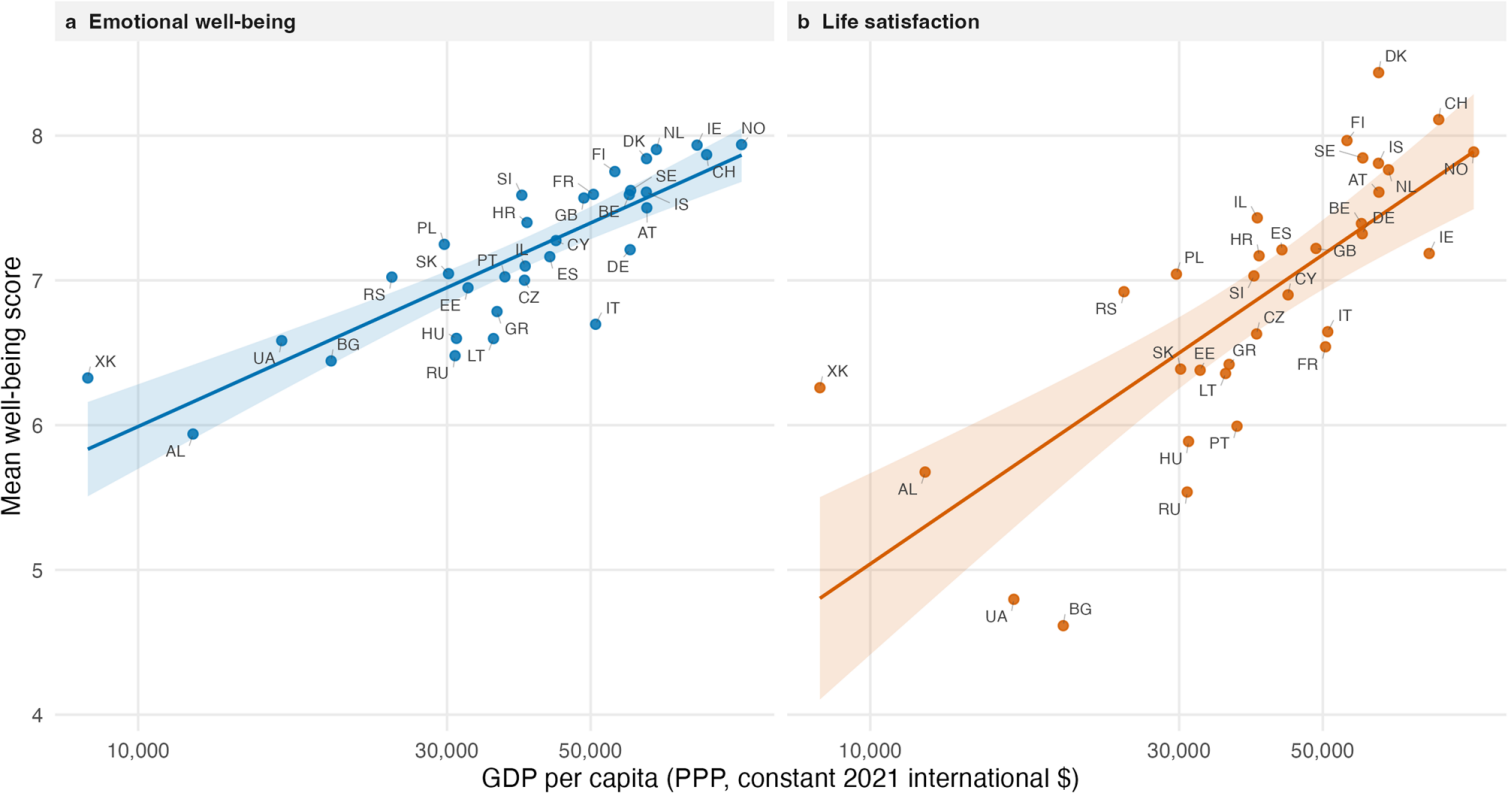
Between-country association between GDP per capita and well-being. Each point is a country-level mean across that country’s observed ESS waves for (**a**) emotional well-being and (**b**) life satisfaction, plotted against the country’s mean log GDP per capita shown on the original GDP scale. Lines are descriptive linear fits on log GDP per capita and shaded areas are 95% confidence intervals.

**Fig. 3:**
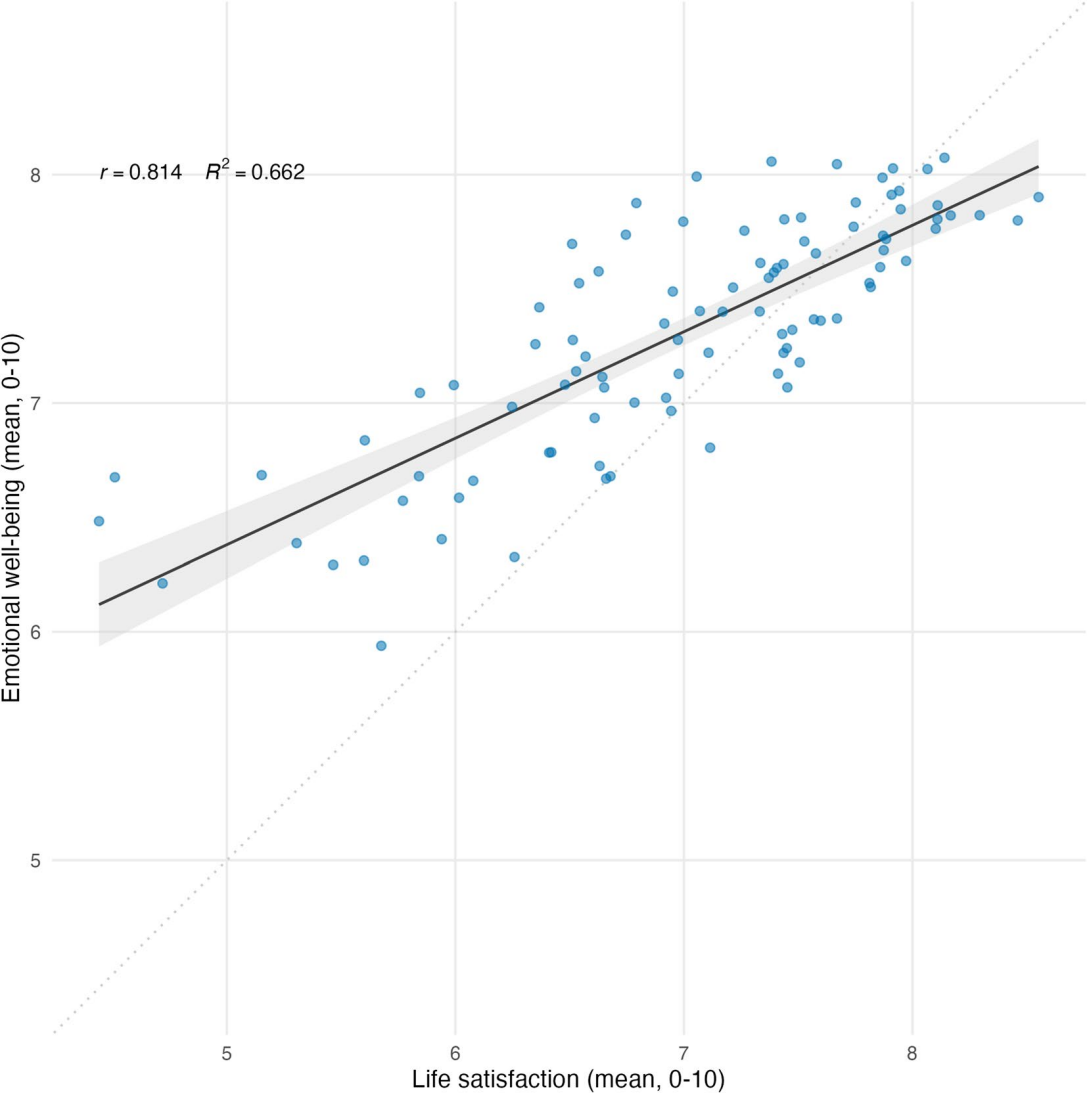
Country-round association between life satisfaction and emotional well-being. Each point is one of the 97 country-rounds. The solid line is a descriptive linear regression of the country-round EWB mean on the LS mean with its 95% confidence interval; the dotted line is the identity y = x for reference. Both measures are on 0–10 scales.

Figure 2 displays the cross-sectional relationships between log real GDP per capita and the two dimensions of subjective well-being using country-level means. Across the 33 country means shown in the figure, GDP is strongly associated with both life satisfaction and emotional well-being. The association is somewhat larger for emotional well-being (*r* = .85, R² = 0.72) than for life satisfaction (*r* = .75, R² = 0.56), although the life-satisfaction slope is steeper because life satisfaction varies more between countries. Life satisfaction and emotional well-being are also strongly related to one another at the country-round level (*r* = .81; Fig. 3), indicating substantial overlap between evaluative and affective well-being.

### Descriptive within-country patterns

**Fig. 4:**
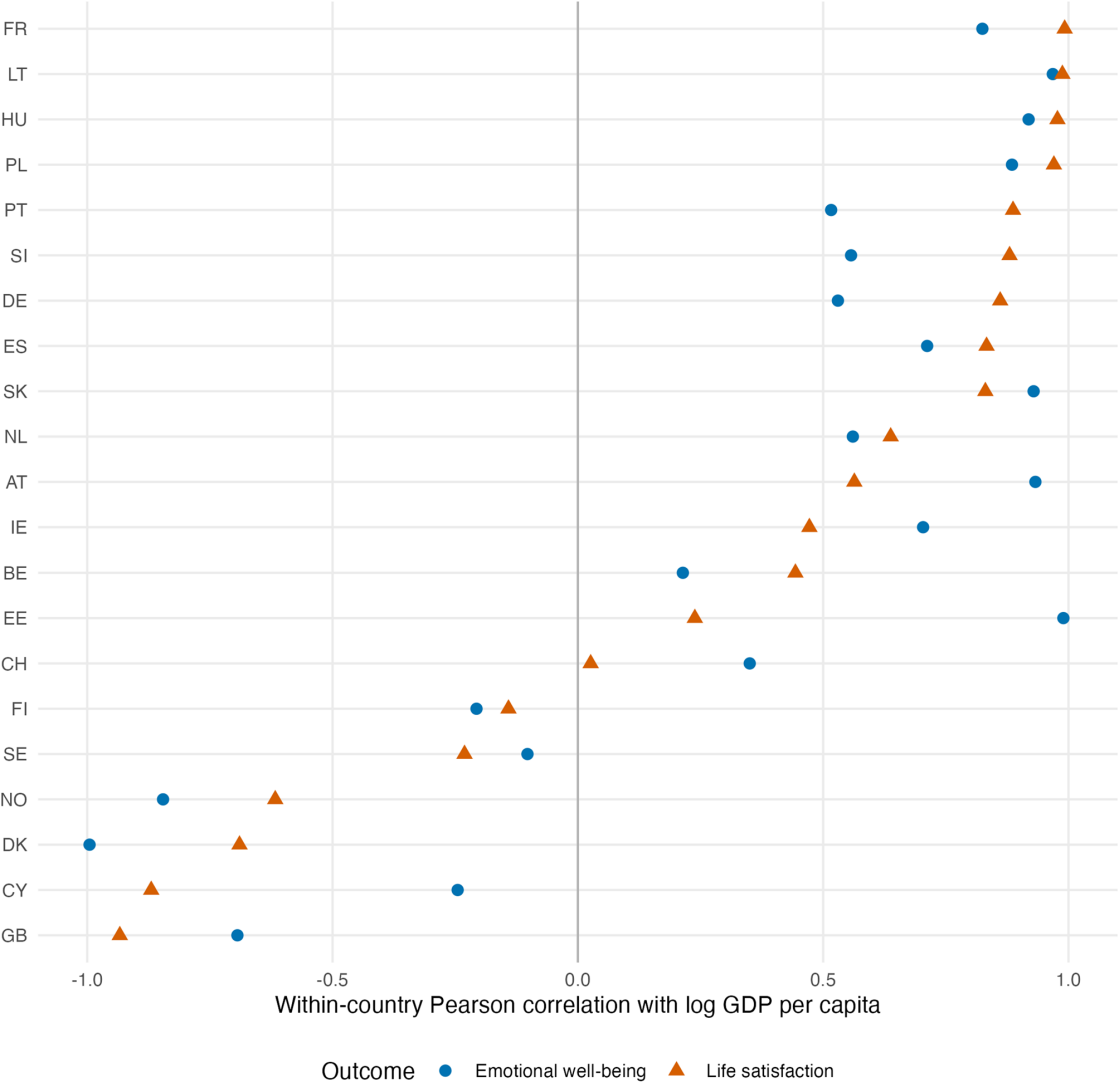
Per-country within-country correlations between log GDP per capita and well-being. Each country contributes one Pearson correlation per outcome (GDP-EWB and GDP-LS), computed from its country-round observations. Restricted to 21 countries with three or more observed ESS waves; countries are ordered by the life-satisfaction correlation, and the vertical reference line marks *r* = 0.

Before turning to the mixed models, Fig. 4 summarizes the country-specific within-country association between log GDP per capita and well-being. Most of the 21 countries with three or more observed ESS waves have positive within-country correlations between GDP and both well-being measures, but several high-income countries, including Denmark, Norway, and the United Kingdom, show negative or near-zero correlations. These descriptive country-specific correlations illustrate heterogeneity around the pooled within-country association and motivate the mixed-model analysis below.

### Mixed-model estimates

We estimated mixed models predicting emotional well-being and life satisfaction from wave indicators, within-country GDP deviations, and between-country GDP differences. Both outcome models used a random country intercept and an AR(1)-type residual structure over survey-wave order (Phi1 = 0.77 for emotional well-being; Phi1 = 0.88 for life satisfaction). Empty random-intercept models yielded high ICCs for both emotional well-being (0.86) and life satisfaction (0.90), indicating that much of the unadjusted country-round variation lies between countries. In the fitted life-satisfaction model, the random-intercept variance was effectively zero, so persistence for that outcome was captured by the AR(1) residual term; the ICCs are therefore reported from intercept-only models to describe the unadjusted multilevel structure.

The emotional well-being model (Table 1) showed positive associations with both within-country GDP (b = 0.92, SE = 0.18, *p* < .001) and between-country GDP (b = 0.88, SE = 0.10, *p* < .001). Net of GDP and relative to 2006, emotional well-being was higher in 2012 (b = 0.13, *p* < .001) and 2014 (b = 0.21, *p* < .001), whereas 2023 did not differ from 2006 (b = 0.01, *p* = .914). Life satisfaction was likewise positively associated with within-country GDP (b = 1.26, SE = 0.35, *p* < .001) and between-country GDP (b = 1.35, SE = 0.21, *p* < .001). None of the life-satisfaction wave indicators was statistically distinguishable from 2006.

**Table 1:** Main models of GDP per capita and well-being.

Predictor	Emotional well-being		Life satisfaction	
	b (SE)	*p*	b (SE)	*p*
Intercept	−2.22 (1.04)*	0.036	−7.41 (2.22)**	0.001
2012 (vs. 2006)	0.13 (0.03)***	< 0.001	0.04 (0.07)	0.579
2014 (vs. 2006)	0.21 (0.04)***	< 0.001	−0.05 (0.09)	0.567
2023 (vs. 2006)	0.01 (0.07)	0.914	−0.02 (0.13)	0.878
GDP within	0.92 (0.18)***	< 0.001	1.26 (0.35)***	< 0.001
GDP within, standardized beta	0.21		0.16	
GDP between	0.88 (0.10)***	< 0.001	1.35 (0.21)***	< 0.001
GDP between, standardized beta	0.73		0.64	

N = 97 country-round observations from 33 countries across four ESS waves (2006, 2012, 2014, 2023/24). GDP is prior-year log real GDP per capita, PPP, constant 2021 international dollars. GDP within and GDP between are Mundlak within-country and between-country components. Cells are unstandardized coefficients with standard errors in parentheses, and p-value columns refer to those unstandardized coefficients. Rows labeled standardized beta are fully standardized GDP coefficients. Both models include a random country intercept and an AR(1)-type residual structure over survey-wave order, estimated by maximum likelihood (Phi1 = 0.767 for emotional well-being; Phi1 = 0.885 for life satisfaction). 2006 is the reference wave. * *p* < .05, ** *p* < .01, *** *p* < .001.

In unstandardized log-GDP units, the within- and between-country coefficients were broadly similar. For emotional well-being, the within-country coefficient was slightly larger than the between-country coefficient (0.92 vs. 0.88); for life satisfaction, the between-country coefficient was slightly larger than the within-country coefficient (1.35 vs. 1.26). Thus, equal proportional GDP contrasts implied broadly comparable well-being differences within and between countries. A 10% higher GDP per capita corresponded to about 0.09 emotional-well-being points and 0.12 life-satisfaction points using the within-country coefficients, and about 0.08 emotional-well-being points and 0.13 life-satisfaction points using the between-country coefficients. A larger cross-national contrast, such as a doubling of GDP per capita, corresponded to approximately 0.61 emotional-well-being points and 0.93 life-satisfaction points using the between-country coefficients.

The fully standardized coefficients are useful as descriptive summaries of the observed panel variation rather than as the primary slope comparison. They were larger for between-country than within-country GDP (emotional well-being: 0.73 vs. 0.21; life satisfaction: 0.64 vs. 0.16) because GDP varied much more between countries than within countries over the observed waves. The standardized coefficients therefore show that observed cross-national GDP differences account for more well-being variation in this panel, not that the unstandardized between-country slope is dramatically stronger.

### Robustness checks and additional specifications

The supplementary analyses supported the main pattern, but they also show where the GDP estimates are most sensitive. Supplementary Tables S1–S6 cover alternative affect scoring, quadratic GDP terms, demographic composition, contextual variables, plausible pathway variables, and asymmetric GDP intervals. Because the macro panel is small and these analyses are closely related, we focus on the stability and direction of the coefficients rather than on isolated p-values.

**Fig. 5:**
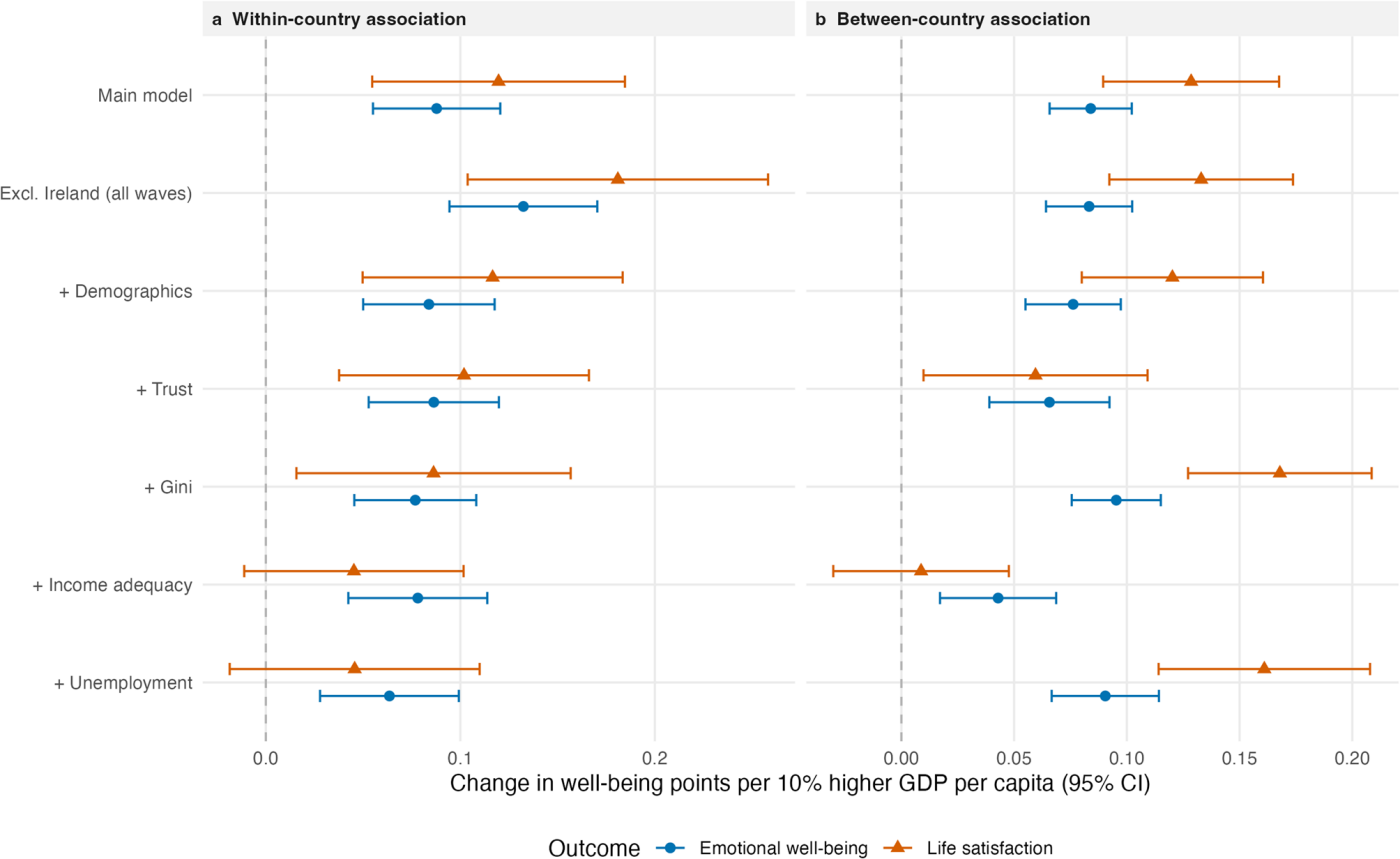
GDP coefficients across specifications. Points and horizontal bars are GDP coefficients with 95% confidence intervals for emotional well-being (blue circles) and life satisfaction (orange triangles), for the (**a**) within-country and (**b**) between-country GDP components, scaled as the expected change in well-being points per 10% higher GDP per capita. The first row is the main model; the second row re-estimates it excluding Ireland from all waves. Each remaining row adds the indicated covariate set to the main model; trust, Gini, income adequacy, and unemployment are entered as within- and between-country components, whereas the demographic-composition row adds percentage female, mean age, and mean years of education.

Figure 5 summarizes these checks on a common scale. It plots the within- and between-country GDP coefficients from the main model and selected alternative specifications as the expected well-being difference associated with 10% higher GDP per capita. One specification excludes Ireland from all waves, since Irish GDP per capita is strongly affected by multinational accounting. The positive GDP associations remained in both the within-country and between-country components after this exclusion. The measurement and specification checks point in the same direction as the main model. When the affect components were modeled separately, GDP was associated with higher positive affect (within b = 1.30, between b = 0.83) and lower negative affect (within b = − 0.57, between b = − 0.95). The emotional-well-being result was therefore not driven by one affect component alone. Quadratic GDP models also improved fit for both outcomes (LRT p values approximately 0.01). The most informative curvature appeared in the within-country component. For emotional well-being, the squared within-country GDP term was negative and significant (b = − 1.45, *p* = .017). Life satisfaction pointed in the same concave direction, but the estimate was less precise (b = − 2.21, *p* = .067). The between-country squared terms were positive rather than saturation-shaped, giving no evidence of diminishing cross-national returns.

The remaining checks concerned sample composition, contextual factors, and candidate pathways. Adjusting for macro-level demographic composition did not materially change the conclusions. Among the contextual factors, income inequality (Gini) left the positive GDP associations intact, whereas social trust attenuated them, particularly between countries. The pathway variables were exploratory. Perceived income adequacy produced the clearest attenuation, absorbing the life-satisfaction association with GDP while the emotional-well-being associations remained positive. Unemployment was more informative for within-country variation and did not account for the between-country life-satisfaction association. These variables are themselves part of broader economic and social development, so the models should be read as robustness and attenuation checks rather than as formal tests of confounding or mediation. Finally, 15 of the 64 observed GDP intervals were declines. The growth-versus-decline analysis did not show clear asymmetry for emotional well-being. For life satisfaction, the decline slope was steeper than the growth slope. This result is suggestive, but it rests on few and mostly modest declines and should not be treated as conclusive.

## Discussion

This study makes three important contributions. First, it shows that economic development is clearly and consistently linked not only to life satisfaction but also to emotional well-being, both across countries and within countries over time. This challenges the common view that income matters mainly for evaluative well-being. Second, it suggests that earlier weak findings for emotional well-being may partly reflect measurement limitations. By using graded, multi-item affect measures with stronger validity, the study shows that emotional well-being exhibits a robust positive association with GDP, highlighting the importance of measurement quality for substantive conclusions. Third, by separating within-country changes from between-country differences in longitudinal mixed models, the article moves beyond static cross-national comparisons and shows that economic conditions are linked to changes in everyday emotional experience. Although we do not claim causality, this design brings the analysis closer to assessing whether economic change is accompanied by change in emotional well-being.

The results therefore qualify a strong version of the view that economic development matters mainly for evaluative well-being. A common expectation is that emotional well-being should be less responsive to economic conditions because affect adapts quickly to improved circumstances^11^, is shaped strongly by stable personality traits^12^, or depends less on the material and opportunity-based considerations that inform global life evaluations^6^. Our findings do not fit this strong version. Emotional well-being was consistently higher in richer countries and at higher GDP levels within countries over the observed waves. This pattern is more consistent with the view that economic development can shape everyday affective experience by improving daily-life conditions, including financial security, exposure to stressors, and access to enjoyable activities^13^. The exploratory attenuation analyses, especially those involving perceived income adequacy and unemployment, are also consistent with this interpretation.

At the same time, the results help explain why previous research has often found weaker associations between national income and affective well-being than between national income and life evaluations^26–28^. This conclusion may depend partly on measurement. Earlier Gallup World Poll studies often relied on dichotomous yesterday-affect items, several of which have limited sensitivity or ambiguous content-validity as measures of emotional valence. In contrast, the present study uses graded affect items with stronger content-validity and prior evidence of cross-national validity^33,34^. This interpretation is consistent with individual-level evidence that the income-affect relationship differs when affect is measured on a sensitive continuous scale rather than with dichotomous items^29^. Under these measurement conditions, emotional well-being is clearly related to economic development. The separate affect analyses further support this interpretation: GDP was associated with both higher positive affect and lower negative affect, suggesting that the association reflects a broader difference in the affective quality of life rather than only one side of the affect balance.

The wave pattern also shows why emotional well-being should be studied directly rather than inferred from life satisfaction. Net of GDP, emotional well-being was higher in 2012 and 2014 than in 2006 and 2023, whereas life satisfaction did not differ across waves once GDP was taken into account. This suggests that the 2023 wave captured period conditions especially relevant to day-to-day emotional experience but not fully captured by GDP per capita or life satisfaction, plausibly including the aftermath of the COVID-19 pandemic and the inflationary period of 2022–2023.

Given the importance of measurement in this study, two issues deserve particular attention. First, the ESS past-week affect items do not provide the same precision as momentary or daily diary measures^38,39^. However, the purpose of the study is not to model fine-grained within-person affective fluctuations, but to compare average emotional well-being across countries and country-rounds. For that purpose, a past-week measure is suitable for large-scale comparative research and is likely less volatile than a one-day snapshot. Second, retrospective emotional well-being reports are sometimes criticized as cognitively contaminated because they correlate more strongly with life satisfaction than momentary measures do. This concern should not be overstated. Berlin and Fors Connolly^10^ showed that lower correlations between momentary affect and life satisfaction partly reflect the low reliability of individual momentary observations. Thus, although life satisfaction and emotional well-being remain conceptually distinct, they are also empirically intertwined.

Several limitations should be acknowledged. First, the design remains observational and country-round based, so the models estimate associations rather than causal effects of GDP per capita. Second, the study covers four ESS waves over 2006–2023, which limits the ability to test adaptation, asymmetric responses to economic decline, and longer-term dynamics. Third, the pathway analyses are exploratory and cannot distinguish mediation from confounding or shared measurement. Finally, the European focus improves comparability of survey measurements and institutional context, but it limits generalizability to poorer countries, rapidly developing economies, and culturally more diverse global samples.

## Conclusion

Taken together, the findings challenge the view that economic development matters mainly for how people evaluate their lives. In this European country-round panel, higher GDP was consistently associated with higher emotional well-being as well as higher life satisfaction, both across countries and within countries over time. The results therefore suggest that economic development is relevant not only to evaluative well-being, but also to the affective quality of everyday life.

## Supplementary Information

Below is the link to the electronic supplementary material.

Supplementary Material 1

## Data Availability

The data that support the findings of this study are openly available from the European Social Survey (ESS) at www.europeansocialsurvey.org. This study uses data from ESS Round 3 (2006), Round 6 (2012), Round 7 (2014), and Round 11 (2023). GDP per capita data are publicly available from the World Bank Open Data repository at data.worldbank.org. Analysis code and data are available on the Open Science Framework (OSF): https://osf.io/5gkp6/overview? view_only=7087592de3ab49c69f75e526639704cf.

## Appendix

**Table A1:** Descriptive statistics for the 97 country-round analytic sample.

Variable	N	M	SD	Min	Max
Life satisfaction (0–10)	97	7.00	0.88	4.44	8.55
Emotional well-being (0–10)	97	7.31	0.51	5.94	8.07
Positive affect (0–10)	97	6.38	0.57	4.94	7.33
Negative affect (0–10)	97	1.76	0.55	0.83	3.30
log real GDP per capita, PPP, constant 2021 international dollars	97	10.70	0.44	9.03	11.72
GDP between (country-mean log GDP component)	97	10.70	0.42	9.03	11.36
GDP within (deviation from country-mean log GDP)	97	-0.00	0.11	-0.32	0.52
% female	97	51.66	1.39	48.45	55.62
Mean age	97	46.76	2.39	38.63	53.77
Mean education years	97	12.55	1.26	7.49	15.94
Perceived household income adequacy (1 very difficult − 4 living comfortably)	97	2.99	0.41	1.86	3.61
Generalized social trust (0–10)	97	5.05	0.93	3.01	6.90
Gini coefficient (WDI)	89	30.98	4.12	23.80	41.30
Unemployment % (WDI)	96	7.90	3.89	2.74	24.79

N = country-round observations (maximum 97). GDP per capita is prior-year real GDP per capita in constant 2021 PPP international dollars; GDP within and GDP between are the Mundlak within-country and between-country components of the log GDP variable. Emotional well-being, positive affect, and negative affect are linearly transformed to 0–10 scales. Perceived household income adequacy is reverse-scored ESS hincfel (higher = household lives more comfortably on present income). Negative affect is not reverse-scored in this table; higher values indicate more negative affect. Gini coefficient and unemployment rate are from the World Bank World Development Indicators and have incomplete coverage.
