# Supplementary material for: Economic development and emotional well-being: longitudinal evidence from 33 European countries
Source: Sci Rep. 2026 Jul 5;16:20568. doi: 10.1038/s41598-026-59695-1 (PMC13333869; doi:10.1038/s41598-026-59695-1)
Supplement: Supplementary file 1 — Supplementary Material 1 [file 41598_2026_59695_MOESM1_ESM.docx]

**Supplementary Information**

**Economic Development and Emotional Well-Being: Longitudinal Evidence from 33 European Countries**

Filip Fors Connolly, Mikael Hjerm and Tommy Gärling

*Supplementary Tables S1-S6*

Supplementary Table S1

*Positive and Negative Affect Modeled Separately*

| **Predictor** | **Positive affect** | **Negative affect** |
| --- | --- | --- |
| 2012 (vs 2006) | 0.20*** (0.05) | -0.08 (0.04) |
| 2014 (vs 2006) | 0.27*** (0.06) | -0.16** (0.05) |
| 2023 (vs 2006) | -0.02 (0.09) | -0.04 (0.08) |
| GDP within | 1.30*** (0.24) | -0.57** (0.20) |
| GDP between | 0.83*** (0.14) | -0.95*** (0.11) |
| *Note. N = 97 country-round observations from 33 countries. Cells are unstandardized coefficients with standard errors in parentheses. Positive and negative affect were linearly rescaled to a 0–10 metric. Negative affect is not reverse-scored in this table; higher values indicate more negative affect. Both models include a random country intercept and an AR(1)-type residual structure over survey-wave order, estimated by maximum likelihood. 2006 is the reference wave. GDP is prior-year log real GDP per capita, PPP, constant 2021 international dollars (World Bank NY.GDP.PCAP.PP.KD); GDP within and GDP between are Mundlak within-country and between-country components. * p < .05. ** p < .01. *** p < .001.* | | |

Supplementary Table S2

*Quadratic GDP Specifications*

| **Predictor** | **Emotional well-being** | **Life satisfaction** |
| --- | --- | --- |
| GDP within | 1.19*** (0.20) | 1.63*** (0.41) |
| GDP within² | -1.45* (0.59) | -2.21 (1.18) |
| GDP between (centered) | 1.05*** (0.14) | 1.83*** (0.28) |
| GDP between² | 0.22 (0.13) | 0.62* (0.27) |
| LRT vs linear (p) | 0.010 | 0.010 |
| *Note. N = 97 country-round observations from 33 countries. Cells are unstandardized coefficients with standard errors in parentheses. Centered quadratic specification: the between-country squared term is built on the country-mean GDP component centered at the sample mean. The likelihood-ratio test compares the quadratic model with the linear model. Both emotional well-being and life-satisfaction models include a random country intercept and an AR(1)-type residual structure over survey-wave order, estimated by maximum likelihood. 2006 is the reference wave. GDP is prior-year log real GDP per capita, PPP, constant 2021 international dollars (World Bank NY.GDP.PCAP.PP.KD); GDP within and GDP between are Mundlak within-country and between-country components. * p < .05. ** p < .01. *** p < .001.* | | |

Supplementary Table S3

*Robustness of GDP Effects to Aggregate Demographic Composition*

|  | **Emotional well-being** | | **Life satisfaction** | |
| --- | --- | --- | --- | --- |
| **Predictor** | **Baseline** | **Adjusted** | **Baseline** | **Adjusted** |
| GDP within | 0.92*** (0.18) | 0.88*** (0.18) | 1.26*** (0.35) | 1.22** (0.36) |
| GDP between | 0.88*** (0.10) | 0.80*** (0.11) | 1.35*** (0.21) | 1.26*** (0.22) |
| % female | — | -0.046 (0.027) | — | -0.171** (0.053) |
| Mean age | — | 0.02 (0.01) | — | -0.03 (0.03) |
| Mean education (yrs) | — | 0.01 (0.03) | — | -0.01 (0.06) |
| *Note. N = 97 country-round observations from 33 countries. Aggregate demographic composition is entered as country-round macro covariates. Cells are unstandardized coefficients with standard errors in parentheses; the %-female coefficient is shown to three decimals. Both emotional well-being and life-satisfaction models include a random country intercept and an AR(1)-type residual structure over survey-wave order, estimated by maximum likelihood. 2006 is the reference wave. GDP is prior-year log real GDP per capita, PPP, constant 2021 international dollars (World Bank NY.GDP.PCAP.PP.KD); GDP within and GDP between are Mundlak within-country and between-country components. * p < .05. ** p < .01. *** p < .001.* | | | | |

Supplementary Table S4

*Robustness of GDP Effects to Contextual Macro Variables (Social Trust and Income Inequality)*

|  | **Emotional well-being** | | | | **Life satisfaction** | | | |
| --- | --- | --- | --- | --- | --- | --- | --- | --- |
| **Predictor** | **Baseline** | **+Trust** | **Baseline (Gini sample)** | **+Gini** | **Baseline** | **+Trust** | **Baseline (Gini sample)** | **+Gini** |
| GDP within | 0.92*** (0.18) | 0.91*** (0.18) | 0.85*** (0.16) | 0.81*** (0.17) | 1.26*** (0.35) | 1.07** (0.34) | 1.13** (0.36) | 0.90* (0.38) |
| GDP between | 0.88*** (0.10) | 0.69*** (0.14) | 1.05*** (0.12) | 1.00*** (0.11) | 1.35*** (0.21) | 0.62* (0.27) | 1.82*** (0.22) | 1.76*** (0.22) |
| Trust within | — | 0.02 (0.08) | — | — | — | 0.31* (0.15) | — | — |
| Trust between | — | 0.14 (0.08) | — | — | — | 0.52*** (0.14) | — | — |
| Gini within | — | — | — | -0.01 (0.01) | — | — | — | -0.05 (0.03) |
| Gini between | — | — | — | -0.03** (0.01) | — | — | — | -0.04 (0.02) |
| *Note. Baseline and +Trust models use N = 97 country-round observations from 33 countries. Baseline (Gini sample) and +Gini models use the Gini-complete sample (N = 89) so Gini attenuation is read within the same sample. Trust and Gini are contextual adjustments rather than definitive confounder controls, and are entered separately for interpretability and to preserve the smaller Gini sample (the cross-country GDP–Gini correlation is weak, r ≈ –.16, so this is not a collinearity concern); attenuation of GDP coefficients reflects overlap with broader social-institutional conditions rather than a clean causal decomposition. Cells are unstandardized coefficients with standard errors in parentheses. Both emotional well-being and life-satisfaction models include a random country intercept and an AR(1)-type residual structure over survey-wave order, estimated by maximum likelihood. 2006 is the reference wave. GDP is prior-year log real GDP per capita, PPP, constant 2021 international dollars (World Bank NY.GDP.PCAP.PP.KD); GDP within and GDP between are Mundlak within-country and between-country components. * p < .05. ** p < .01. *** p < .001.* | | | | | | | | |

Supplementary Table S5

*Exploratory Attenuation of the GDP Coefficients by Candidate Pathways (Perceived Household Income Adequacy and Unemployment)*

| **Outcome** | **Predictor** | **Baseline** | **+ Perceived income adequacy** | **Baseline (unemployment sample)** | **+ Unemployment** |
| --- | --- | --- | --- | --- | --- |
| Emotional well-being | GDP within | 0.92*** (0.18) | 0.82*** (0.19) | 0.92*** (0.17) | 0.67*** (0.19) |
|  | GDP between | 0.88*** (0.10) | 0.45** (0.14) | 0.99*** (0.11) | 0.95*** (0.13) |
|  | Perceived income adequacy within | — | 0.31 (0.18) | — | — |
|  | Perceived income adequacy between | — | 0.64*** (0.16) | — | — |
|  | Unemployment within | — | — | — | -0.02** (0.01) |
|  | Unemployment between | — | — | — | -0.02 (0.02) |
| Life satisfaction | GDP within | 1.26*** (0.35) | 0.48 (0.30) | 1.22*** (0.35) | 0.48 (0.34) |
|  | GDP between | 1.35*** (0.21) | 0.09 (0.21) | 1.71*** (0.22) | 1.69*** (0.25) |
|  | Perceived income adequacy within | — | 1.71*** (0.28) | — | — |
|  | Perceived income adequacy between | — | 1.94*** (0.24) | — | — |
|  | Unemployment within | — | — | — | -0.05*** (0.01) |
|  | Unemployment between | — | — | — | -0.01 (0.04) |
| *Note. The baseline column uses N = 97 country-round observations from 33 countries and is the correct comparison for perceived income adequacy, which is complete for all country-rounds. The Baseline (unemployment sample) and +Unemployment columns use the unemployment-complete sample (N = 96), so unemployment attenuation is read within the same sample. Each candidate pathway is added separately to its baseline (columns are not cumulative). These are exploratory pathway/attenuation checks, not formal mediation analyses: perceived income adequacy is evaluatively close to subjective well-being, and unemployment may act as a stressor/mediator, or contextual shock. Cells are unstandardized coefficients with significance stars and standard errors in parentheses; — = term not in the model. Both emotional well-being and life-satisfaction models include a random country intercept and an AR(1)-type residual structure over survey-wave order, estimated by maximum likelihood. 2006 is the reference wave. GDP is prior-year log real GDP per capita, PPP, constant 2021 international dollars (World Bank NY.GDP.PCAP.PP.KD); GDP within and GDP between are Mundlak within-country and between-country components. * p < .05. ** p < .01. *** p < .001.* | | | | | |

Supplementary Table S6

*Asymmetry of Well-being Changes With Respect to GDP Rises and Declines (Split-Slope Specification)*

|  | **Pooled across intervals** | | | **Period-adjusted (interval fixed effects)** | | |
| --- | --- | --- | --- | --- | --- | --- |
| **Outcome** | **Rise slope** | **Decline slope** | **Wald p (rise vs decline)** | **Rise slope** | **Decline slope** | **Wald p (rise vs decline)** |
| Emotional well-being change | 0.25 (0.22) | 1.74 (2.15) | 0.504 | 0.81*** (0.19) | 3.33* (1.65) | 0.137 |
| Life satisfaction change | 1.11*** (0.32) | 8.03* (3.17) | 0.035 | 1.07** (0.35) | 8.48** (3.04) | 0.018 |
| *Note. Within-country first differences in log real GDP per capita (constant 2021 PPP) split into rises (d_up = max(Δ log GDP, 0)) and declines (d_down = min(Δ log GDP, 0)). The pooled specification regresses each well-being change on d_up and d_down; the period-adjusted specification adds interval fixed effects to absorb pan-European shocks. Of 64 observed within-country intervals, 15 were real-GDP declines (49 were rises); the declines ranged in magnitude from less than 0.1% to 5.9% in cumulative GDP change between consecutive ESS waves, with the largest decline observed for IE 2006-2012. The Wald p reflects the null of equal rise- and decline-slopes, computed from the full coefficient covariance matrix. Cells are unstandardized slope coefficients with significance stars and standard errors in parentheses. Loss-aversion asymmetry is suggestive for life satisfaction (steeper decline-slope, p < .05 under both specifications) but absent for emotional well-being, and rests on few decline intervals; treat as exploratory. * p < .05. ** p < .01. *** p < .001.* | | | | | | |
